# Extremism and common mental illness: cross-sectional community survey of White British and Pakistani men and women living in England

**DOI:** 10.1192/bjp.2019.14

**Published:** 2020-10

**Authors:** Kamaldeep Bhui, Michaela Otis, Maria Joao Silva, Kristoffer Halvorsrud, Mark Freestone, Edgar Jones

**Affiliations:** 1 Professor of Cultural Psychiatry and Epidemiology, Centre for Psychiatry, Wolfson Institute of Preventive Medicine, Queen Mary University of London; and Honorary Consultant Psychiatrist, East London NHS Foundation Trust, UK; Head of Centre for Psychiatry and Director of Collaborating Centre, World Psychiatric Association; 2 Statistician, Centre for Psychiatry, Wolfson Institute of Preventive Medicine, Barts and The London School of Medicine and Dentistry, Queen Mary University of London, UK; 3 Research Fellow, Centre for Psychiatry, Wolfson Institute of Preventive Medicine, Barts and The London School of Medicine and Dentistry, Queen Mary University of London, UK; 4 Senior Lecturer, Centre for Psychiatry, Wolfson Institute of Preventive Medicine, Barts and The London School of Medicine and Dentistry, Queen Mary University of London, UK; 5 Professor in History of Medicine and Psychiatry, Institute of Psychiatry, Psychology and Neuroscience, King's College London, UK

**Keywords:** Depression, dysthymia, extremism and radicalisation, sociocultural

## Abstract

**Background:**

Mental illnesses may explain vulnerability to develop extremist beliefs that can lead to violent protest and terrorism. Yet there is little evidence.

**Aims:**

To investigate the relationship between mental illnesses and extremist beliefs.

**Method:**

Population survey of 618 White British and Pakistani people in England. Extremism was assessed by an established measure of sympathies for violent protest and terrorism (SVPT). Respondents with any positive scores (showing sympathies) were compared with those with all negative scores. We calculated associations between extremist sympathies and ICD-10 diagnoses of depression and dysthymia, and symptoms of anxiety, personality difficulties, autism and post-traumatic stress. Also considered were demographics, life events, social assets, political engagement and criminal convictions.

**Results:**

SVPT were more common in those with major depression with dysthymia (risk ratio 4.07, 95% CI 1.37–12.05, *P* = 0.01), symptoms of anxiety (risk ratio 1.09, 95% CI 1.03–1.15, *P* = 0.002) or post-traumatic stress (risk ratio 1.03, 95% CI 1.01–1.05, *P* = 0.003). At greater risk of SVPT were: young adults (<21 versus ≥21: risk ratio 3.05, 95% CI 1.31–7.06, *P* = 0.01), White British people (versus Pakistani people: risk ratio 2.24, 95% CI 1.25–4.02, *P* = 0.007) and those with criminal convictions (risk ratio 2.23, 95% CI 1.01–4.95, *P* = 0.048). No associations were found with life events, social assets and political engagement.

**Conclusion:**

Depression, dysthymia and symptoms of anxiety and post-traumatic stress are associated with extremist sympathies.

## Violence prevention and public health

Terrorist incidents are common in countries such as Afghanistan, Iraq, Pakistan and Syria. Although they are rarer in Western Europe, North America and other high-income countries, they still have devastating health and societal consequences globally.^1^^,^^2^ Those who commit acts of terrorism are hypothesised as having been through a process of radicalisation, defined as the adoption of extreme political or ideological attitudes.^3^^,^^4^ In high-income countries, radicalisation and terrorist offending are largely managed by criminal justice agencies, although public mental health interventions are now proposed as having preventive value.^5^^–^^7^ Public health approaches to understanding and preventing radicalisation require better evidence of the risk factors associated with the adoption of extremist attitudes and terrorist behaviour more generally.^8^^,^^9^ Indeed, in the UK, public servants including doctors and mental health professionals are asked to show ‘due regard’ to the identification of those at risk of radicalisation.^3^

Extremist views and attitudes are more common than acts of terrorism, and may indicate a preliminary stage of the radicalisation process that can be prevented.^5^^,^^8^^,^^10^ Research into violence prevention, especially in relation to terrorist offending, presents a significant ethical challenge. Violence prevention in general and countering violent extremism within a public health framework requires a different type of population science and cycles of learning to implement and test favoured theories in research and actual practice.^5^^,^^7^ Therefore, this study adopts a population approach to better understand the drivers of radicalisation and extremist attitudes more generally, and the links with symptoms of psychological and mental illnesses.

## Mental illness and extremism

The literature on the links between mental illness and violent radicalisation specifically, and extremism more generally, is sparse. Many terrorist offenders do not have mental illnesses or criminal histories,^8^ but recent policy and research invokes a link between mental illnesses – specifically depression, psychoses and autism – with the risk of radicalisation and terrorist offending.^11^^,^^12^ Findings from our previous survey reported links between extremist sympathies and depressive symptoms rather than ICD-10 (1992) diagnoses which we now include. We also showed that being under the age of 25, being born in the UK, having fewer social contacts or considering religion were important risk factors.^13^ Depressive symptoms explained a significant proportion of the association between life events and political engagement with extremist sympathies.^14^ Building on our previous studies of extremist beliefs and depressive symptoms in Pakistani and Bangladeshi men and women, this article presents the findings from a new population cohort that compares Pakistani and White British people, who were assessed for ICD-10 diagnoses of depressive illness and of dysthymia rather than depressive symptoms. We also assessed symptoms of personality disorders, autism, generalised anxiety and post-traumatic stress disorder (PTSD). These additional symptoms were included to tackle speculation of relevance, despite there being little empirical evidence.^13^^,^^14^ Our new analyses attempted to replicate previous work on different samples, with better measures of depressive diagnoses and extremism. We again considered other factors such as social, political and cultural influence which have been proposed as risk factors of extremist attitudes.^3^^,^^8^^,^^13^^–^^15^

## Methods

### Participants

We recruited 618 men and women, aged 18–45 years, of White British and Pakistani heritage living in the UK community in three locations: Blackburn with Darwen, Bradford and Luton. Quota sampling was applied to yield equal numbers from each location (*n* = 206 each) and equal numbers of Pakistani and White British respondents overall (*n* = 309 each). For analyses, these numbers were weighted by the demographic frequencies in the location. UK Census data were used to identify a specified geographical area (called a Lower Layer Super Output Area) with higher proportions of residents of Pakistani heritage. These areas were used as sampling locations. Equal quotas were also set for age (18–30 years and 31–45 years), gender and work status (working full-time, not working full-time). The survey was delivered through Ipsos MORI Social Research Institute. Trained and locally based interviewers worked to a structured invariant interview format and to industry standards under supervision, offering language matching if required. Individuals within sampling locations were recruited by door knocking, and were interviewed after seeking informed consent. A handheld computer and flash cards were used to simplify the process of answering multiple-choice and sensitive questions, and to reduce social-desirability bias. The variables and measures were identified and prepared by the research team (K.B. and E.J.) and Ipsos MORI, and refined in pilot testing and cognitive debriefs before launching the survey. Ethical approval was granted by Queen Mary University of London Research Ethics Committee on 19 November 2015: QMERC2015/06.

### Measures

#### Psychiatric variables

ICD-10 depression diagnosis was measured using the Clinical Interview Schedule – Revised (CIS-R),^16^ using the following symptoms: depression experienced most days, most of the time, for at least two weeks was classified into ‘mild’ (four symptoms), ‘moderate’ (five to six symptoms) and ‘severe’ (seven or more symptoms). We define ‘major depression’ as either moderate or severe depression diagnoses. A diagnosis of depression required at least one symptom of persistent sadness/low mood, loss of interest/pleasure or fatigue/low energy; as well as problems with sleep, concentration, confidence, appetite, suicidality, agitation or guilt/self-blame. This measure was scored using well-established algorithms.^17^ To correct for missing data (*n* = 137, 22.2%) on depression symptoms, 122 respondents were diagnosed by consensus ratings made between two clinicians, and if necessary a third, reviewing all the survey data leading to 98% completion on depression diagnosis (*n* = 603).

Dysthymia (i.e. persistent mild depression, or depressive personality) was measured by using seven characteristic symptoms, each rated on presence, persistence and longevity. Symptoms consisted of: feeling depressed, inadequate, effort in everything, unable to cope with everyday demands, unable to enjoy anything, trouble sleeping and complaining/moaning. Using the ICD-10 criteria for dysthymia, a binary diagnosis was assigned with two or more depressive symptoms experienced ‘often’ or ‘always’ (i.e. to meet the criteria ‘most of the days; more days than not’), and lasting ‘for more than 2 years’.^18^ To minimise missing data on dysthymia (*n* = 70, 11.3%), 67% of these were completed from rerouted items in the CIS-R, Patient Health Questionnaire (PHQ-9) and the PTSD Checklist (PCL-C) (for rerouting methods, see Supplementary Table 1 available at https://doi.org/10.1192/bjp.2019.14).^16^^,^^19^^,^^20^

We constructed a combined depression–dysthymia variable that distinguished those with comorbid major depression with dysthymia (*n* = 23, 4%) from diagnoses of dysthymia only, major depression only, mild depression only and neither.

Autism symptoms were measured by using a total score on the Autism Spectrum Quotient (AQ-10), which is reported as having high discriminant validity for those with and without a clinical diagnosis.^21^

Personality disorder symptoms were measured by using the total score on the Standardised Assessment of Personality – Abbreviated Scale (SAPAS),^22^ which consists of eight indicator questions about the presence of maladaptive personality traits. SAPAS was reported to have high diagnostic validity. The sum of maladaptive traits was used rather than a threshold of four or more to reflect a greater risk of ‘any’ personality disorder.

PTSD was measured by using the civilian PCL-C consisting of 17 items, which was reported to have high diagnostic validity.^19^ Symptom clusters included re-experiencing, avoidance or numbing, and arousal. Response options were: 1 = ‘not at all’, 2 = ‘a little bit’, 3 = ‘moderately’, 4 = ‘quite a bit’ and 5 = ‘extremely’; scores of three or more were considered symptomatic. Three items that had been omitted to avoid repetition were rerouted from alternative survey items; these consisted of ‘irritability/anger’ and ‘exaggerated startle’ from the Generalised Anxiety Disorder scale (GAD-7), and ‘concentration difficulty’ from the CIS-R.^16^^,^^23^ Rerouted items were retained after sensitivity analyses produced similar results. The total PTSD score was used rather than the diagnostic threshold of 30 or more.

The GAD-7 is a well-established measure of anxiety symptoms across seven items.^23^ Each item was scored on a four-point Likert scale consisting ‘not at all’, ‘several days’, ‘more than half days’ and ‘nearly every day’; the total score was entered into the analyses. The GAD-7 is routinely used in mental health and primary care services. We used the total score.

Alcohol consumption, illicit substance use and tobacco use were assessed using a binary yes/no response after asking about lifetime consumption.

#### Social variables

A criminal conviction was scored as a binary yes/no response (at least one conviction) for any offence in the Gunn criminal profile.^24^

A measure of discrimination was adopted from the EMPIRIC study.^25^ This asked about experiences of physical assault, property damage, insults, unfair treatment at work and job refusal due to race, religion or culture; each item scored 0–5 (total score of 0–25).

Life events were measured using the 12-item List of Threatening Experiences (scored 0–12).^26^

As a measure of social support, we asked about the number of contacts by telephone, email or visit in the preceding 2 weeks by friends or relatives. Low social capital has been associated with violence,^27^^,^^28^ suicide^29^ and poor mental health.^30^ In accord with previous research, we selected questions from the Office for National Statistics Social Capital Question Bank^31^ to ask about satisfaction with living in the area (very satisfied, fairly satisfied, neither, fairly dissatisfied or very dissatisfied), trust in neighbours (many people, some people, a few or none) and feelings of safety (very safe, fairly safe, fairly unsafe or very unsafe). Scores were summed (ranging from 3–13).

Political engagement items were drawn from the Department of Communities and Local Government Citizenship Survey.^32^ We asked whether individuals had voted in the past local council election, discussed politics or political news with someone else, signed a petition, donated money to a charity or campaigning organisation, paid a membership fee to a charity or campaigning organisation, undertaken voluntary work, boycotted certain products (for political, ethical or environmental reasons), boycotted certain products for religious reasons, expressed political opinions online, been to any political meeting, donated money or paid membership fees to a political party and taken part in a demonstration, picket or march. Yes/no responses were summed (ranging from 0–12).

#### Sympathies for violent protest and terrorism

Radicalisation risk was identified using a tool called the ‘SyfoR’: Sympathies for Radicalisation.^13^^–^^15^ The sympathies for violent protest and terrorism (SVPT) tool was originally developed using participatory discussions with Muslim and non-Muslim researchers and community panels in local mental health charities, educational organisations and religious institutions for improved content validity and readability. We asked for feedback on the study design and findings from our independent scrutiny committee comprising two professors of marketing and communications, a professor of business studies and a senior academic psychologist.

The SyfoR has been independently reviewed as having high content, criterion and construct validity; internal consistency; readability and low respondent burden.^33^ The tool was updated to include one additional item – the act of going to Syria to fight with Islamic State, leading to a total of 17 items (see Supplementary Table 2). Participants were asked to rate each item using a seven-point Likert scale ranging from −3 (completely condemn) to 3 (completely sympathise); a score of 0 represented neither condemnation nor sympathy (interpreted as neutral).

The SVPT measure used in this study was developed following a principal components analysis on the 17 SyfoR items. Seven items were found to have a distinct latent structure related to SVPT (see Supplementary Table 2). These items comprised sympathising with (a) committing minor crime, (b) committing violence… in political protests, (c) organising radical terrorist groups, (d) threatening to commit terrorist actions, (e) committing terrorist actions… as a form of political protest, (f) using bombs and (g) using suicide bombs to fight against injustices. The omitted SyfoR items related to latent factors comprising defensive violence, UK foreign policy and fighting against British troops. Respondents were categorised as sympathisers (any positive scores), condemners (all negative scores) or neutral (any neutral score, without any positive scores). Neutral scores are presented in the descriptive and univariate analyses. In the multivariable models we present the risk of sympathisers, with condemners as the comparison group.

### Statistical analyses

A principal components factor analysis with orthogonal rotation on the 17 SVPT items identified a four-factor model (see Supplementary Table 1), retaining factors using the Kaiser criterion, and confirmed using parallel analysis. Factor one – a seven-item structure titled ‘political violence and terrorism’ – was retained due to its distinct structure, which produced moderate/strong inter-item correlations (*r* = 0.43–0.86), strong inter-item reliability (Cronbach's α = 0.91), appropriate sampling adequacy (Kaiser-Meyer-Olkin = 0.862), explained variance (σ^2^ = 67%) and was split-half validated. Following sensitivity analyses, respondents were categorised into three mutually exclusive groups (as described above) using scores on the seven items. Missing responses on SVPT items were conservatively treated as scores less than zero. Sensitivity analyses tested alternative computations of SVPT – namely with missing responses treated as values of one, values of zero or omitted.

The total weighted prevalence (for categorical variables) and weighted means and standard errors (for continuous variables) were tabulated by the three groups: those showing sympathies, condemners and the rest.

Each demographic, social and psychiatric variable was entered into a univariate analyses, using multinomial logistic regression with the three SVPT groups as the outcome. In univariate analyses, we compared associations among sympathisers, and those neutral, with the condemners as the reference group. Sensitivity analyses compared the findings when using threshold and total scores for psychiatric measures.

In multivariable analyses, associations were then assessed among sympathisers as our main outcome of interest, using condemners as the comparison group. Psychiatric variables were entered in stepwise regression analyses to test for individual and multivariable effects on our main variables of interest – comorbid major depression and dysthymia – and then followed by symptoms of autism, personality disorder and PTSD. Anxiety symptoms were not entered with PTSD symptoms in the same model due to collinearity. Otherwise, all variables showing significant associations in univariate analyses were retained for multivariate models. Multivariable analyses were conducted with and without imputing missing data for anxiety and PTSD symptoms. Sensitivity analyses compared multivariable models by using complete cases and imputed data.

All analyses were conducted using Stata version 13.1 for Windows. The regression models and frequency estimates were weighted using the ‘pw’ weight command, which adjusted for the quota sample, and non-response to ensure the findings were representative of the population. Mediation analyses were conducted using the ‘ldecomp’ command. The ‘mi’ command imputed PTSD and anxiety data, using all other variables in the multivariable model, to produce ten imputed data sets.

The main findings were sustained in sensitivity analyses when testing alternative computations of SVPT; when conventional diagnostic thresholds were applied to generate binary variables for PTSD, autism and personality disorder; and when multivariable analyses were conducted on complete cases rather than imputed data. Correlations between psychiatric disorders were moderate to strong (*r* = 0.35–0.74, *P* < 0.01) and this informed our multivariable modelling approach.

## Results

### Sample characteristics: the role of sociocultural, demographic and lifestyle factors

**Fig. 1:**
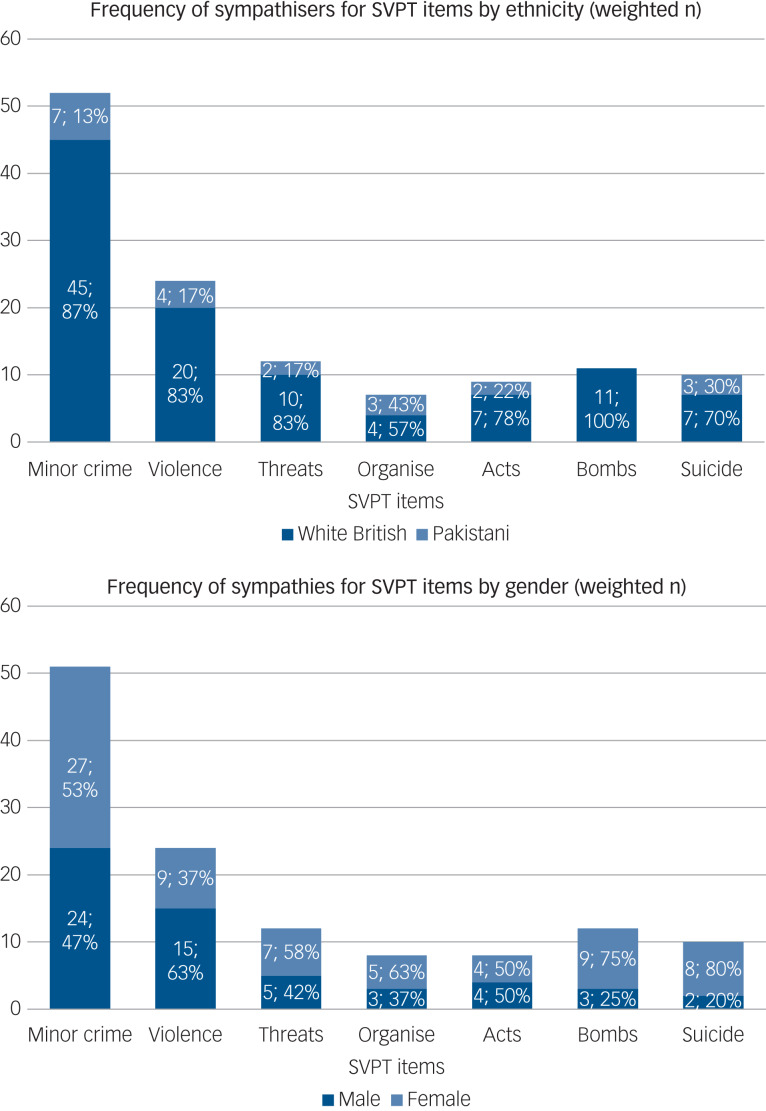
Seven items of the SVPT measure: endorsement by gender and ethnicity.

The survey identified 341 (61%) respondents who condemned violent protest and terrorist actions, 144 (26%) were neutral and 73 (13%) were shown to have SVPT. SVPT were shown by 15.1% of the White British and 8.1% of the Pakistani groups (see Table 1 and Fig. 1). SVPT were significantly more common in lifetime alcohol drinkers, tobacco users, illicit drug users and in those with a criminal conviction (Tables 1 and 3). Younger people, single people and those born in the UK more often expressed SVPT as compared with older, married or divorced people and those born outside the UK. Gender, religion, religious attendance, education level, political engagement, life events, discrimination, social capital and social support were not associated with SVPT, and were therefore not entered in the multivariable analyses.

**Table 1:** Demographic, social and psychiatric characteristics by sympathies for violent protest and terrorism groups (weighted)

Variables	Condemners (*n* = 341, 61%)	Neutral (*n* = 144, 26%)	Sympathisers (*n* = 73, 13%)	Total (*n* = 558, 100%)
Age groups				
18–20	29 (41.0)	25 (35.2)	17 (23.8)	71 (100)
21–25	42 (54.3)	27 (34.9)	8 (10.8)	77 (100)
26–30	64 (60.2)	29 (27.6)	13 (12.2)	106 (100)
31–35	59 (69.1)	19 (22.0)	7 (8.9)	85 (100)
36–40	63 (63.4)	24 (24.5)	12 (12.1)	99 (100)
41–45	72 (70.7)	17 (16.7)	13 (12.7)	102 (100)
Gender				
Male	177 (64.4)	61 (22.2)	37 (13.4)	275 (100)
Female	164 (57.8)	83 (29.3)	36 (12.9)	283 (100)
Marital status				
Single	158 (52.6)	89 (29.7)	53 (17.7)	300 (100)
Married	162 (70.4)	51 (22.3)	17 (7.3)	230 (100)
Separated/divorced	21 (77.2)	3 (10.2)	3 (12.5)	27 (100)
Income				
Less than £5000	42 (56.5)	20 (26.9)	13 (16.6)	75 (100)
£5000–£14 999	59 (54.6)	28 (26.5)	20 (18.9)	107 (100)
£15 000–£24 999	61 (64.5)	22 (23.4)	12 (12.1)	95 (100)
£25 000–£34 999	43 (62.7)	17 (23.9)	9 (13.4)	69 (100)
£35 000 or more	42 (69.7)	12 (19.7)	6 (10.6)	60 (100)
Employment				
Employed	243 (65.2)	85 (22.9)	45 (11.9)	373 (100)
In education	25 (49.8)	18 (35.0)	8 (15.3)	51 (100)
Unemployed	27 (48.3)	20 (34.9)	9 (16.8)	56 (100)
Retired/housewife/sick	45 (59.2)	20 (26.6)	11 (14.2)	76 (100)
Education				
No qualifications	15 (46.2)	12 (37.9)	5 (15.9)	32 (100)
GCSE/CSE/A Level	208 (58.5)	100 (28.3)	47 (13.2)	355 (100)
Bachelor/Master/PhD	112 (70.7)	27 (16.9)	19 (12.4)	158 (100)
Place of birth				
UK	284 (58.2)	136 (27.9)	68 (14.0)	488 (100)
Pakistan	35 (81.0)	6 (12.7)	3 (6.3)	44 (100)
Ethnicity				
White	236 (57.9)	110 (27.0)	61 (15.1)	407 (100)
Pakistani	105 (69.6)	34 (22.3)	12 (8.1)	151 (100)
Location^a^				
Blackburn	108 (55.5)	56 (28.7)	31 (15.8)	195 (100)
Bradford	117 (61.5)	53 (27.5)	21 (11.0)	191 (100)
Luton	115 (67.0)	35 (20.5)	22 (12.5)	172 (100)
Religion				
None	85 (56.9)	50 (33.8)	14 (9.3)	149 (100)
Christian	124 (59.1)	49 (23.4)	37 (17.5)	210 (100)
Buddhist	–	1 (100)	–	1 (100)
Muslim	99 (67.5)	36 (24.3)	12 (8.3)	147 (100)
Atheist	23 (70.8)	3 (11.3)	6 (17.9)	32 (100)
Other	2 (24.6)	1 (20.3)	4 (5.5)	7 (100)
Religious attendance				
Never	171 (55.7)	97 (31.7)	38 (12.5)	306 (100)
Monthly or less	101 (66.2)	29 (18.9)	23 (15.0)	153 (100)
Weekly or more	69 (69.8)	18 (17.8)	12 (12.4)	99 (100)
Alcohol consumption				
No	97 (63.5)	43 (28.2)	13 (8.4)	153 (100)
Yes	244 (60.2)	101 (24.8)	60 (15.0)	405 (100)
Illicit drug use				
No	265 (61.6)	120 (28.1)	44 (10.3)	429 (100)
Yes	68 (58.3)	21 (18.6)	27 (23.1)	116 (100)
Any criminal conviction				
No	311 (62.6)	125 (25.2)	60 (12.2)	496 (100)
Yes	30 (52.8)	14 (24.3)	13 (22.9)	57 (100)
Tobacco use				
No	160 (67.2)	56 (23.7)	22 (9.2)	238 (100)
Yes	176 (55.8)	87 (27.8)	52 (16.4)	315 (100)
Other demographics, mean (standard error)				
Discrimination	0.24 (0.04)	0.27 (0.06)	0.17 (0.06)	0.24 (0.03)
Political engagement	3.51 (0.14)	2.90 (0.21)	3.99 (0.39)	3.32 (0.11)
Life events score	1.06 (0.89)	0.91 (0.12)	1.37 (0.20)	1.05 (0.07)
Social support	6.33 (0.14)	6.00 (0.24)	5.82 (0.36)	6.10 (0.11)

a. The weighted number of respondents by location was 154 (79%) White British to 41 (21%) Pakistani in Blackburn; 137 (72%) White British to 54 (28%) Pakistani in Bradford; and 116 (67%) White British to 56 (33%) Pakistani in Luton.

### Common mental disorders

SVPT were positively associated with a diagnosis of comorbid major depression and dysthymia (compared with those with neither diagnosis), and with PTSD and anxiety symptoms (Table 2). There were too few participants with SVPT and major depression (*n* = 4) or SVPT and mild depression (*n* = 2) to assess the influence of these single diagnoses.

**Table 2:** Mental disorders and sympathies for violent protest and terrorism

SVPT categories	Condemners (*n* = 341, 61%)	Neutral (*n* = 144, 26%)	Sympathisers (*n* = 73, 13%)	Total (*n* = 558, 100%)	Missing (*n* = 60, 10%)
ICD-10 mood disorders, *n* (%)					
None	272 (61.0)	120 (27.0)	53 (12.0)	445 (100)	84 (13.59)
Mild depression	21 (68.2)	8 (25.2)	2 (0.07)	31 (100)	
Dysthymia alone	9 (48.4)	4 (22.5)	5 (29.1)	18 (100)	
Major depression alone	15 (70.8)	2 (11.8)	4 (17.5)	21 (100)	
Major depression and dysthymia	10 (44.6)	5 (19.8)	8 (35.6)	23 (100)	
Symptom scores, mean (standard error)					
Autism score	2.47 (0.09)	2.50 (0.15)	2.76 (0.21)	2.51 (0.07)	78 (12.62)
Personality disorder score	2.46 (0.08)	2.45 (0.13)	2.61 (0.18)	2.48 (0.07)	84 (13.59)
PTSD score	26.8 (0.76)	25.8 (1.13)	32.2 (1.80)	27.3 (0.60)	89 (14.40)
GAD-7 score	3.07 (0.28)	3.19 (0.47)	5.28 (0.75)	3.41 (0.24)	108 (17.5)

SVPT, sympathies for violent protest and terrorism; ICD-10, International Classification of Diseases 10th revision; PTSD, post-traumatic stress disorder; GAD-7, Generalised Anxiety Disorder.

**Table 3:** Univariate logistic regressions using seven-item sympathies for violent protest and terrorism as an outcome

Participant characteristics	Risk ratio (95% CI)	*P*	*N*
Age group: years (reference group: 18–20)			539
21–25	0.34 (0.12, 0.94)	0.04	
26–30	0.35 (0.14, 0.88)	0.03	
31–35	0.22 (0.08, 0.66)	0.001	
36–40	0.33 (0.12, 0.90)	0.03	
41–45	0.31 (0.12, 0.79)	0.02	
Gender (reference group: male)			558
Female	1.07 (0.60, 1.91)	0.81	
Marital status (reference group: single)			557
Married	0.31 (0.16, 0.61)	0.001	
Divorced/separated	0.48 (0.16, 2.17)	0.34	
Income (reference group: <£5000 per year)			413
£5000–14 999 per year	1.18 (0.50, 2.78)	0.70	
£15 000–24 999 per year	0.64 (0.24, 1.65)	0.35	
£25 000–£34 999 per year	0.73 (0.25, 2.13)	0.56	
£35 000 or more per year	0.52 (0.15, 1.77)	0.56	
Employment (reference group: employed)			555
In education	1.68 (0.68, 4.15)	0.26	
Unemployed	1.91 (0.81, 4.50)	0.14	
Retired/housewife/sickness	1.31 (0.61, 2.82)	0.49	
Education (reference group: no qualifications)			546
GCSE/CSE/A Level	0.66 (0.22, 1.97)	0.45	
Bachelor/Master/PhD	0.51 (0.15, 1.68)	0.27	
Place of birth (reference group: UK)			535
Pakistan	0.33 (0.11, 0.94)	0.04	
Ethnicity (reference group: Pakistani)			558
White British	2.24 (1.25, 4.02)	0.007	
Religion (reference group: none)			547
Christian	1.80 (0.80, 4.07)	0.16	
Buddhist	–	–	
Muslim	0.75 (0.33, 1.71)	0.49	
Atheist	1.55 (0.42, 5.74)	0.52	
Other	–	–	
Religious attendance (reference group: never)			558
Monthly or less	1.01 (0.52, 1.94)	0.99	
Weekly or more	0.79 (0.37, 1.69)	0.54	
Alcohol consumption	1.89 (1.01, 3.54)	0.048	558
Illicit substance use	2.37 (1.25, 4.48)	0.008	549
Tobacco use	2.15 (1.19, 3.90)	0.01	553
Criminal conviction: any	2.23 (1.01, 4.95)	0.048	553
Discrimination	0.82 (0.56, 1.21)	0.32	531
Political engagement	1.08 (0.96, 1.22)	0.22	558
Life events	1.13 (0.96, 1.33)	0.13	558
Social support	0.92 (0.81, 1.03)	0.16	558
Depression/dysthymia (reference group: none)			534
Mild depression	0.50 (0.10, 2.61)	0.41	
Dysthymia only	3.06 (0.86, 10.88)	0.08	
Major depression only	1.26 (0.34, 4.65)	0.73	
Major depression and dysthymia	4.07 (1.37, 12.05)	0.01	
Autism score	1.13 (0.94, 1.35)	0.19	540
Personality disorder score	1.08 (0.89, 1.30)	0.46	534
PTSD score, 17-item	1.03 (1.01, 1.05)	0.003	529
GAD-7 score	1.09 (1.03, 1.15)	0.002	507

Condemners used as reference group; weighted, unadjusted values. PTSD, post-traumatic stress disorder; GAD-7, Generalised Anxiety Disorder.

Autism and personality disorder scores were not associated with SVPT. However, the individual item in the personality schedule of ‘losing one's temper easily’ was positively associated with SVPT (risk ratio 2.25, 95% CI 1.12–4.53, *P* = 0.02, *n* = 530).

### Multivariable associations with extremist sympathies

In multivariable analyses, symptoms of PTSD and anxiety and ICD-10 diagnoses of major depression with dysthymia were positively associated with SVPT, after adjusting for age, ethnicity, marital status and criminality (Table 4, model 1). Neither autism nor personality disorder was associated with SVPT. Throughout these models, younger age remained positively associated with SVPT. SVPT were more common in people with a criminal conviction, smokers and single people, with non-significant positive association with the White British compared with the Pakistani group. When adjusting for PTSD symptoms, anxiety symptoms and substance use, the association between sympathies and comorbid major depression and dysthymia was diminished (Table 4, models 2–4), indicating that PTSD and anxiety symptoms accounted for the association between comorbid major depression and dysthymia with SVPT. This was sustained when adjusted for age, ethnicity, marital status and criminality.

**Table 4:** Stepwise, multivariable and multinomial regression for seven-item sympathies for violent protest and terrorism

	Model 1: Comorbid DD (*n* = 510, *F* = 2.37)^a^		Model 2: Comorbid DD, and PTSD^b^ (*n* = 510, *F* = 2.37)		Model 3: Comorbid DD, and anxiety^b^ (*n* = 508, *F* = 2.08)		Model 4: Comorbid DD and substances (*n* = 500, *F* = 2.74)	
Risk of SVPT (ref: condemners)	Risk ratio (95% CI)	*P*	Risk ratio (95% CI)	*P*	Risk ratio (95% CI)	*P*	Risk ratio (95% CI)	*P*
Risk factors/confounders								
Comorbid major depression and dysthymia (ref: none)	3.50 (1.12, 10.93)	0.03	2.02 (0.37, 10.96)	0.41	1.75 (0.38, 8.09)	0.47	2.53 (0.75, 8.50)	0.13
PTSD score^b^			1.02 (0.99, 1.05)	0.30				
Anxiety score^b^					1.07 (0.98, 1.16)	0.11		
Tobacco use (ref: no): yes							2.60 (1.23, 5.51)	0.01
Illicit drug use (ref: no): yes							1.39 (0.63, 3.07)	0.41
Alcohol use (ref: yes): no							0.46 (0.12, 1.76)	0.26

Condemners used as reference group; weighted, multiply imputed values. Adjusted for age, ethnicity, marital status and criminal convictions. DD, major depression and dysthymia; PTSD, post-traumatic stress disorder; SVPT, sympathies for violent protest and terrorism; ref, reference group.

a. Adjusting for income reduced the sample size (*n* = 326); therefore, income was omitted from all models. Income diminished the significance of depression.

b. Anxiety was entered as an alternative to PTSD due to collinearity. The results for tobacco, illicit drug and alcohol use in model 4 were sustained when anxiety or PTSD were also entered into the model.

## Discussion

This is the first empirical evidence to link common mental disorders with extremist sympathies in populations of both White British and Pakistani men and women living in England. The association between extremist sympathies and comorbid depression and dysthymia was explained by underlying severe anxiety and PTSD symptoms. A more general approach to improving population mental health alongside prevention in specific populations such as those experiencing post-traumatic symptoms and younger people may be helpful. A previous study of teenage boys in Gaza also indicated the importance of mood symptoms, although the depressive experiences were particularly severe and related to the immediacy of violence related to war and conflict, making it difficult to consider the sources of depression to be similar in such vastly different settings.^34^ Our findings on depression are at variance with studies of violence in gang members and of pro- and anti-British attitudes; depression was negatively associated with violence, but positively associated with anxiety and with traumatic experiences.^35^^,^^36^ Our findings may be explained by the combination of dysthymia – a chronic condition that depletes hope and capability to overcome adversity – and depression. Or the underlying dysthymia alone may be more important, as we found positive but non-significant trends for an association.

Surprisingly, extremist sympathies were more prevalent in White British than in Pakistani people. Less surprising is the higher prevalence in single people, in those with a criminal conviction and lifetime users of tobacco, alcohol and illicit drugs. Although these lifestyle factors suggest personality function may be relevant, we found no associations with personality disorders.

These findings are consistent with a study of terrorist offenders in the US, which reported that propensity to extremist political violence was greater in those with a criminal history, a mental illness diagnosis or suspected mental illness, alcohol and drug use, and a history of trauma.^37^ The US study found that propensity was higher in those with experiences of community marginalisation, measured as perceived imminent threat from an external group, political crisis, collective crisis situation or group-facilitated cognition. In contrast, we did not find that social influences or assets (i.e. social capital, social contacts or discrimination) were related to extremist sympathies. The differences might be explained by the setting, namely a study of offenders in the US in contrast to our population sample. However, in contrast to our present study and consistent with the US study, our previous study using a different measure of extremism did show fewer social contacts in those with extremist sympathies and a lower risk associated with social assets.^13^

Previous research on psychological risk factors for violent extremism have discussed concepts of mindset- and world view-related violence.^38^ Volitional incompetence is described as an affective deficit leading to increased receptivity to extreme ideologies such as dogmatic, fundamentalist, authoritarian or apocalyptic world views. Our results support evidence for a volitional propensity related to low self-regulation and self-control as a risk of extremism, with the association between sympathies and mood disorders, PTSD, anxiety and poor impulse control (losing one's temper). A psychotherapeutic clinical trial improving volitional competence led to reduction in depressive symptoms.^39^ However, our findings also suggest anxiety and post-traumatic symptoms underlie the associations with depression and dysthymia, suggesting complex and common mental states are responsible rather than single illnesses. ^40^

Public agencies are asked to show due regard to the recognition and prevention of extremist offending, although this proposal is controversial.^12^ The link between mental illness and extremist attitudes is proposed to be higher in lone actors, than in group-based terrorism.^11^ This suggests that, in the absence of links with extremist groups or histories of extremist offending, the presence of mental illnesses may add risk. In conclusion, the exacerbating role of depression is proposed to be cross-culturally relevant, as are other mood disorders that might indicate an affective vulnerability, specifically dysthymia alone or with depression are important correlates of sympathies.

### Strengths and weaknesses

The strengths of this study include using ICD-10 diagnostic algorithms for depression and dysthymia, which was an advance on previous studies. We used validated scalar measures of psychiatric symptoms for PTSD, generalised anxiety disorder, autism and personality disorder; all shown to have strong validity but of course risk false positive and false negatives. Also, we did not assess specific personality disorders such as antisocial, histrionic or obsessive–compulsive personality disorders.^41^ The distinction between outright sympathisers of extremism, those neutral and outright condemners allowed for the characterisation of those most at risk with those least at risk. We could have compared the most extreme with the neutral responders, but we were not able to clarify why people answered the way they did; we could not judge whether neutral responders were truly neutral or did not want to commit a response. Hence we excluded them from the final analyses.

A potential limitation of the SVPT measure is that respondents need only sympathise with one item to be considered to hold extreme views. This was to maximise power and had face validity by separating out all positive, neutral and negative scores. Using a threshold of two or more sympathies to classify sympathisers led to consistent point estimates, although the power was compromised with only 23 people then showing sympathies. Sympathising with committing minor crime was one item that was the most commonly endorsed. Excluding this item from the classification of those with sympathies produced no major changes in point estimates. A comprehensive approach would be to test these associations in a psychiatric population in future studies that also assess associations with violence.

A recent review of mental disorders and terrorist involvement reported that empirical research is often reductionist in its dichotomy of terrorists versus non-terrorists.^42^ Our study responds to this by analysing a preliminary phase that is distinct from acts of violent extremism: how those who sympathise with political acts of violent radicalisation and terrorism differ from those who condemn such acts. Yet radicalisation processes are proposed to act on these very people to adopt extremist views; hence our comparisons can indicate which characteristics are associated with the adoption of such views. Travelling to foreign lands to fight and religious ideologies were not central to the measure of extremism that we used, so those interested in specific ideologies that we did not measure may prefer an alternative assessment method that may be unique to one specific set of extremist ideologies. In this article we used a measure that was valuable across ethnic and religious groups and the same seven items were used for both ethnic groups.

Measuring extremist behaviour and engagement with extremist networks is complex; thus measuring sympathies for such acts provides a way of studying a potential susceptibility to violent behaviour and terrorism ethically, without incrimination or breaches of confidentiality. We cannot infer a more significant link to extremist attitudes on our measures and actual future violent behaviours at this stage. We are testing the measures further with violent and non-violent offender patients.

Recruiting people to such a study is not straightforward. The use of quota sampling was an efficient recruitment method, which matched sampling areas to the target population using census data; it is often used in market research and national surveys where no listing of eligible participants exists.^43^^,^^44^ Given the sensitivity of the survey topic, quota sampling avoided exposing large numbers of people who would not have met the inclusion criteria to the preliminary recruitment phase. Yet, reassuringly, our estimates of prevalence for depression and dysthymia were consistent with other published studies.

## Supplementary material

For supplementary material accompanying this paper visit https://doi.org/10.1192/bjp.2019.14. click here to view supplementary material
