# Supplementary material for: Extremism and common mental illness: cross-sectional community survey of White British and Pakistani men and women living in England
Source: Br J Psychiatry. 2020 Oct;217(4):547–54. doi: 10.1192/bjp.2019.14 (PMC7525107; doi:10.1192/bjp.2019.14)
Supplement: Supplementary file 1 [file S000712501900014Xsup001.docx]

| **Not for publication**   \| ***Supplementary Table 1. Rerouting methods for dysthymia symptoms from CISR, PHQ and PTSD items*** \| \| \| --- \| --- \| \| ***Dysthymia symptom: Tiredness/depression*** \| \| \| ***A score of:***  ***CISRB1: Have you noticed that you have been getting tired in the past month? [YES]***  ***And at least one of:***  ***CISRB4: In the past seven days, including (DAY OF WEEK), on how many days have you felt tired/lacking in energy?[4 days or more]***  ***or***  ***CISRB5: Have you felt tired/lacking in energy for more than 3 hours in total on any day in the past week? Please exclude time spent sleeping.[YES]***  ***And:***  ***CISRB9: How long have you been feeling tired/lacking in energy in the way you have just described? [2 years or more]*** \| ***Tired/depressed symptom = present*** \| \| ***At score of:***  ***CISRG1: Almost everyone becomes sad, miserable or depressed at times. Have you had a spell of feeling sad, miserable or depressed in the past month? [YES]***  ***Or***  ***In the past week have you had a spell of feeling sad, miserable or depressed? [YES]***  ***And at least one of:***  ***CISRG6: Since last (CURRENT DAY OF WEEK), on how many days have you felt sad, miserable or depressed/unable to enjoy take an interest in things? [4 YEARS OR MORE]***  ***Or***  ***CISRG7: Have you felt sad, miserable or depressed/unable to take an interest in things for more than 3 hours in total on any day in the past week?***  ***And:***  ***CISRG10: How long have you been feeling sad, miserable or depressed/unable to take an interest in things in the way you have described? [2 YEARS OR MORE]*** \| ***Tired/depressed symptom = present*** \| \| ***Dysthymia symptom: Effort in daily activities*** \| \| \| ***A score of:***  ***CISRB2: During the past month, have you felt you’ve been lacking in energy? [YES]***  ***Or***  ***CISRB6: Have you felt so tired/lacking in energy that you’ve had to push yourself to get things done during the past week? [YES]***  ***And:***  ***CISRB4: In the past seven days, including (DAY OF WEEK), on how many days have you felt tired/lacking in energy?[4 days or more]***  ***or***  ***CISRB5: Have you felt tired/lacking in energy for more than 3 hours in total on any day in the past week? Please exclude time spent sleeping.[YES]***  ***And:***  ***CISRB9: How long have you been feeling tired/lacking in energy in the way you have just described? [2 years or more]*** \| ***Effort symptom = present*** \| \| ***Dysthymia symptom: Trouble sleeping*** \| \| \| ***A score of:***  ***CISRB3a What is the main reason [for tiredness]? Can you choose from the list below? [sleep]***  ***And at least one of:***  ***CISRB4: In the past seven days, including (DAY OF WEEK), on how many days have you felt tired/lacking in energy?[4 days or more]***  ***or***  ***CISRB5: Have you felt tired/lacking in energy for more than 3 hours in total on any day in the past week? Please exclude time spent sleeping.[YES]***  ***And:***  ***CISRB9: How long have you been feeling tired/lacking in energy in the way you have just described? [2 years or more]*** \| ***Sleep symptom = present*** \| \| ***A score of:***  ***CISRD1: In the past month, have you been having problems with trying to get to sleep or with getting back to sleep if you woke up or were woken up? [YES]***  ***And at least one of:***  ***CISRD3: On how many of the past seven nights did you have problems with your sleep?[4 nights or more]***  ***Or***  ***CISRD6: In the past week, on how many nights did you spend 3 or more hours trying to get to sleep?[4 nights or more]***  ***Or***  ***CISRD7: Do you wake more than two hours earlier than you need to and then find you can’t get back to sleep? [YES]***  ***And:***  ***CISRD10: How long have you been having the problems with sleep as you have described? [2 years or more]*** \| ***Sleep symptom = present*** \| \| ***Dysthymia symptom: Not able to enjoy anything*** \| \| \| ***A score of:***  ***CISRB7: Have you felt tired/lacking in energy when doing things that you enjoy during the past week? [YES]***  ***And:***  ***CISRB4: In the past seven days, including (DAY OF WEEK), on how many days have you felt tired/lacking in energy?[4 days or more]***  ***or***  ***CISRB5: Have you felt tired/lacking in energy for more than 3 hours in total on any day in the past week? Please exclude time spent sleeping.[YES]***  ***And:***  ***CISRB9: How long have you been feeling tired/lacking in energy in the way you have just described? [2 years or more]*** \| ***Enjoyment symptom = present*** \| \| ***A score of:***  ***CISRG2: During the past month, have you been able to enjoy or take an interest in things as much as you usually do? [NO]***  ***And at least one of:***  ***CISRG6: Since last (CURRENT DAY OF WEEK), on how many days have you felt sad, miserable or depressed/unable to enjoy take an interest in things? [4 days or more]***  ***And:***  ***CISRG10: How long have you been feeling sad, miserable or depressed/unable to take an interest in things in the way you have described? [2 years or more]*** \| ***Enjoyment symptom = present*** \| \| ***Dysthymia symptom: Feel tired and depressed***  ***[Symptoms are indicated as present with responses: all the time/often, and indicated as absent for responses: sometimes/rarely/never]*** \| \| \| ***If dysthymia is missing, then absence in both of the following items, or one if the other is missing, qualifies for an absent symptom:***  ***PHQ9A: Feeling tired or having little energy [NOT AT ALL] *note: “several days” and “more than half days” may indicate presence of symptom.***  ***And/or***  ***PHQ9C: Feeling down, depressed, or hopeless [NOT AT ALL] *note: “several days” and “more than half days” may indicate presence of symptom.*** \| ***Tired/depressed symptom = absent*** \| \| ***Dysthymia symptom: Trouble sleeping***  ***[Symptoms are indicated as present with responses: all the time/often, and indicated as absent for responses: sometimes/rarely/never]*** \| \| \| ***If dysthymia is missing, then absence in both of the following items, or one if the other is missing, qualifies for an absent symptom:***  ***Q50M (PTSD): Trouble falling or staying asleep? [NOT AT ALL-MODERATELY] *”quite a bit” and “extremely” may indicate presence of symptom.***  ***And/or***  ***PHQ9D: Trouble falling or staying asleep or sleeping too much [NOT AT ALL]. *note: “several days” and “more than half days” may indicate presence of symptom.*** \| ***Sleep symptom = absent*** \| \| ***Dysthymia symptom: Not able to enjoy anything***  ***[Symptoms are indicated as present with responses: all the time/often, and indicated as absent for responses: sometimes/rarely/never]*** \| \| \| ***If dysthymia is missing, then absence in both of the following items, or one if the other is missing, qualifies for an absent symptom:***  ***Q50_i: Loss of interest in things that you used to enjoy? [NOT AT ALL-MODERATELY] *”quite a bit” and “extremely” may indicate presence of symptom.***  ***And/or***  ***PHQ9_B: Little interest or pleasure in doing things (e.g. hobbies) [NOT AT ALL] *note: “several days” and “more than half days” may indicate presence of symptom.*** \| ***Enjoyment symptom = absent*** \| \| ***Dysthymia symptom: feel inadequate***  ***[Symptoms are indicated as present with responses: all the time/often, and indicated as absent for responses: sometimes/rarely/never]*** \| \| \| ***If dysthymia is missing, then absence in the following item qualifies for an absent symptom:***  ***PHQ9_G: Feeling bad about yourself – or that you are a failure or have let yourself or your family down [NOT AT ALL] *note: “several days” and “more than half days” may indicate presence of symptom.*** \| ***Inadequate symptom = absent*** \|   **Supplementary Table 2.** Rotated factor loadings for 17 items measuring sympathies for violent protest and terrorism (SVPT) (obs=507, 82%) | | | | |
| --- | --- | --- | --- | --- | --- | --- | --- | --- | --- | --- | --- | --- | --- | --- | --- | --- | --- | --- | --- | --- | --- | --- | --- | --- | --- | --- | --- | --- | --- | --- | --- | --- | --- | --- | --- | --- | --- | --- | --- | --- | --- | --- | --- | --- |
| **Sympathies for Violent Protest and Terrorism items** | **Factor 1 Political violence and terrorism** | **Factor 2 Defensive violence** | **Factor 3 British citizens fighting UK** | **Factor 4 Foreign policy** |
| Take part in a non-violent political protests | -0.3687 | **0.4607** | -0.0161 | -0.0568 |
| Commit minor crime in political protests | **0.4036** | **0.5096** | 0.1016 | -0.0688 |
| Use violence in political protests | **0.7396** | 0.3460 | 0.1186 | -0.0361 |
| Threaten to commit terrorist actions as a form of political protest | **0.8583** | 0.1342 | 0.2584 | -0.0211 |
| Organize radical terrorist groups but do not personally participate in protests or violence | **0.8061** | 0.0647 | 0.3797 | -0.0604 |
| Commit terrorist actions as form of political protest | **0.8419** | 0.0751 | 0.3289 | -0.0422 |
| The use of bombs to fight against injustices | **0.6935** | 0.1508 | 0.3206 | -0.1342 |
| The use of suicide bombs to fight against injustices | **0.7773** | 0.0807 | 0.3088 | -0.0912 |
| The use of violence to protect your family | -0.2741 | **0.6374** | -0.0279 | -0.2945 |
| The use of violence by organised groups to protect people of their own race group | 0.2132 | **0.7114** | 0.1893 | -0.0759 |
| The use of violence to fight against injustice by the police | 0.2503 | **0.7907** | 0.1516 | -0.0433 |
| The use of violence to fight against injustice by the government | 0.2476 | **0.8096** | 0.193 | -0.0371 |
| Went to Afghanistan to fight against UK and other international forces | 0.2693 | 0.1158 | **0.9071** | -0.0274 |
| Went to Iraq to fight against UK and other international forces | 0.2411 | 0.1130 | **0.9269** | -0.0429 |
| Have gone to Syria to fight with Islamic State against international forces | 0.3741 | 0.1111 | **0.8125** | -0.0881 |
| The UK government deciding to send military forces to Syria *(reverse coded)* | -0.0618 | -0.1053 | -0.0298 | **0.9254** |
| The government's decision to send UK soldiers to Iraq *(reverse coded)* | -0.0792 | -0.0349 | -0.0831 | **0.9228** |
